# Medical students’ perspectives on racism in medicine and healthcare in Germany: Identified problems and learning needs for medical education

**DOI:** 10.3205/zma001604

**Published:** 2023-04-17

**Authors:** Simon Matteo Gerhards, Mark Schweda, Merle Weßel

**Affiliations:** 1 Carl von Ossietzky University of Oldenburg, School of Medicine and Health Sciences, Department for Health Services Research, Medical Ethics Division, Oldenburg, Germany

**Keywords:** racism, healthcare, medical education, medical students, Germany

## Abstract

**Objective:**

Against the backdrop of considerable lack of research, this study provides the first exploration of medical students’ perspectives on racism in medicine and healthcare in Germany. The aim is to identify problems and learning needs for medical education. We address the following research questions:

– How do medical students perceive racism in medicine and healthcare in Germany?

– How do they address, understand, and discuss different aspects of racism in this context?

– What are their expectations regarding the role of medical education?

**Methods:**

Semi-structured online focus group discussions were conducted with 32 medical students from 13 different medical schools in Germany. The discussions were transcribed and analyzed using qualitative content analysis.

**Results:**

Based on the analysis of the focus groups, four main hypotheses could be formulated:

1. Medical students perceive racism in medicine and healthcare in Germany as a ubiquitous phenomenon.

2. They have problems to identify racist behaviour and structures due to conceptual knowledge gaps.

3. They are insecure how to deal with racism on a situational level.

4. They hold medical education accountable to tackle racism in medicine and healthcare on various levels.

**Conclusion:**

Our study raises specific learning needs for addressing racism in medicine and healthcare in Germany. Research from the US-context might inspire innovative approaches for German medical education but needs to take national specificities into account. Further research is needed to prepare the implementation of antiracist training in German medical education.

## Zusammenfassung

**Zielsetzung:**

Vor dem Hintergrund erheblicher Forschungslücken bietet diese Studie die erste Exploration der Perspektiven von Medizinstudierenden auf Rassismus in Medizin und Gesundheitsversorgung in Deutschland. Ziel ist die Identifikation von Problemen und Lernbedarfen für die medizinische Ausbildung. Wir gehen den folgenden Forschungsfragen nach:

– Wie nehmen Medizinstudierende Rassismus in Medizin und Gesundheitsversorgung in Deutschland wahr?

– Wie adressieren, verstehen und diskutieren sie verschiedene Aspekte von Rassismus in diesem Kontext?

– Welche Erwartungen haben sie an die medizinische Ausbildung?

**Methoden:**

Es wurden sechs semi-strukturierte Online-Gruppendiskussionen mit insgesamt 32 Medizinstudierenden von 13 verschiedenen medizinischen Fakultäten in Deutschland durchgeführt. Die Diskussionen wurden aufgezeichnet, transkribiert und mit der Methode der qualitativen Inhaltsanalyse ausgewertet.

**Ergebnisse:**

Im Zuge der Analyse der Gruppendiskussionen wurden vier Hypothesen formuliert:

1. Rassismus in Medizin und Gesundheitsversorgung wird von den Medizinstudierenden als ubiquitäres Phänomen wahrgenommen.

2. Sie haben aufgrund begrifflicher Unklarheiten Probleme, rassistisches Verhalten und rassistische Strukturen zu erkennen.

3. Sie zeigen Unsicherheiten im situativen Umgang mit Rassismus.

4. Die medizinische Ausbildung trägt für sie Verantwortung zur Bekämpfung von Rassismus auf allen Ebenen von Medizin und Gesundheitsversorgung.

**Schlussfolgerung:**

Unsere Studie zeigt spezifische Probleme und Lernbedarfe hinsichtlich des Umgangs mit Rassismus in Medizin und Gesundheitsversorgung auf. Forschung aus dem US-amerikanischen Kontext kann innovative Ansätze für die medizinische Ausbildung in Deutschland vermitteln, jedoch müssen nationale Besonderheiten berücksichtigt werden. Weitere Forschung sollte die Implementierung von rassismuskritischer Lehre in der deutschen medizinischen Ausbildung begleiten.

## 1. Introduction

In the revision of the *National Competence Based Catalogue of Learning Objectives for Undergraduate Medical Education* (NKLM) [https://www.nklm.de], racism is explicitly addressed as a relevant topic of German medical education for the first time (e.g., NKLM 2.0, VIII.6-04.4.13) [https://nklm.de/zend/objective/view/id/10001324/essential/yes/lve/211449]. This marks the acknowledgment of a long-standing desideratum in Germany. The rejection of any form of discrimination constitutes an important precept of medical ethics articulated in prominent professional codices, such as the World Medical Association's Geneva Declaration. Moreover, at the national level, the German General Act on Equal Treatment (AGG) also applies to racist discrimination in the healthcare system [1]. Nevertheless, the topic of racism has long been neglected in German medical education and has only partially been discussed from a historical perspective focusing on medicine under National Socialism [2]. In the wake of contemporary antiracist movements, professional networks and student initiatives highlight the gap between the national discussion and the state of the international debate [3], [4], [5], [6].

Especially within the Anglo-American discussion, there has been a surge of research and strategic actions addressing racism, discrimination, and structural disparities in medicine and healthcare in recent years [7], [8]. By comparison, the state of the German debate is underdeveloped, and at best, fragmentary [9]. Although several studies highlight differences in healthcare and health status of migrant populations [10] and report experiences of racist discrimination [11], [12], comprehensive data on, and detailed analyses of, racism in medicine and healthcare in Germany are largely missing [13]. One of the consequences is the lack of empirical knowledge and evidence-based, context-sensitive approaches to the problem in German medical education. Isolated studies have analyzed the subtle influence of professional socialization on the ways in which medical students in Germany deal with socio-cultural diversity and interculturality [14]. However, there is not currently any systematic research explicitly examining their perspectives on racism. Thus, research that provides reliable knowledge on the actual state of their relevant competences and learning needs in this field is urgently needed, in order for suitable didactical strategies to realize the related learning objectives and address racism in medicine and healthcare.

Against this backdrop, our study provides the first exploration of medical students’ awareness of racism in medicine and healthcare in Germany. Guided by Kern’s six step approach of curriculum development [15], we identify problems and learning needs for medical education. Our research questions are:

- How do medical students perceive racism in medicine and healthcare in Germany?
- How do they address, understand, and discuss different aspects of racism in this context?
- What are their expectations regarding the role of medical education?

To answer these questions, we conducted online focus groups with students from medical schools all over Germany. The discussions covered aspects of interpersonal, institutional, and structural racism as well as demands for medical education to address these topics. The data were analyzed with qualitative content analysis.

## 2. Methods

Given the lack of previous research on this topic, we developed an explorative qualitative study. To elucidate medical students’ awareness and understanding of racism in medicine and healthcare, we conducted several moderated online focus group discussions with four to seven participants. This method appeared particularly suitable to elicit a range of different positions and arguments. 

The recruitment of participants took place between June and August 2021. Public calls for participation were disseminated via local student bodies, offices of student affairs, lecturers, informal student groups, and social media. We used a snowball sampling method. A pre-questionnaire was distributed to collect socio-demographic information (age, gender, study year, experience of discrimination, political activities). 

The composition of the sample was aimed to be as diverse as possible regarding gender, study year, sociocultural background, and geographical location. Inclusion criteria were legal age, sufficient proficiency in the German language, and enrolment in medicine at a university in Germany. Participants provided written informed consent in advance. Institutional review board approval was obtained from the ethics committee of the Medical Faculty of the University of Oldenburg (No. 2021-080).

Altogether, six focus group discussions with 32 participants took place between July and September 2021. The participants came from 13 different medical schools across Germany (see table 1 (Tab. 1)). The group discussions were conducted online with the video conference tool Webex and lasted between 90 minutes and two hours. The discussions were moderated by a team of two facilitators, a medical student at the University of Oldenburg and a post-doctoral researcher who teaches medical ethics at the same institution. They used a semi-structured discussion guideline which covered experiences and understandings of interpersonal, institutional, and structural racism as well as students’ possible demands regarding medical education. The audio from the discussions was recorded, and then transcribed verbatim and pseudonymized. We conducted a structured qualitative content analysis following Kuckartz, assisted by MAXQDA software [16]. For this purpose, we derived a set of codes to structure the material according to central thematic aspects. In addition, inductive codes were created during the analysis process to capture themes emerging from the material. The analysis resulted in the identification of four hypotheses.

## 3. Results

Our results illustrate medical students’ multi-layered perceptions of racism in medicine and healthcare in the German context. They mirror their problem awareness as well as their difficulties in addressing issues of racism. Thus, they also point to specific learning needs and perspectives on the curriculum in medical education. 

### 3.1. The experience of racism is ubiquitous in medicine

In the focus group discussions, racism in medicine and healthcare appears as an omnipresent problem that links interpersonal interaction with institutional and structural factors. 

#### 3.1.1. Racism in interindividual interactions

Throughout all the conducted focus groups, medical students report racism in interindividual interactions within healthcare and medical education, irrespectively of different medical specialties. While racist discrimination from healthcare workers against patients is perceived as particularly problematic, experiences of racism are reported across all groups in medicine, healthcare, and education, such as patients, doctors, nurses, lecturers, and students. One student who identifies as a person of colour in the discussion explains: *“as a patient or a medical professional, as a person of colour in a hospital, you just expect to experience racism”* (F5,27). Racism is a common experience for racialized healthcare practitioners and students. For example, they describe being perceived as less competent, not as a doctor (D1,64), or being referred to as “unclean” and carriers of infectious diseases (C3,23). Some patients refused to be examined by them (D3,23). The combination of subtle everyday experiences of racism and explicit racist aggressions, such as somebody who* “throws a T-shirt at me and says, well, my T-shirt comes from where you come from”* (F5,27) are seen as troubling and impeding the affected students’ focus and learning experience: *“that’s just fucking exhausting to sit there all the time and justify your existence”* (F5,27).

##### 3.1.2. Institutionalized racism: racist knowledge (gaps) in medicine

Racism is also reported in context of institutional and structural conditions. In medical schools, students remember racist knowledge and stereotypes that are imparted from academic and clinical teaching. They criticize lecturers’ lack of sensitivity for, and the uncritical use of, racist stereotypes and categorizations:

*“the lecturer had projected a small comic on the wall, where […] somehow a room was to be seen. A turban wearing man with a long beard and a bomb belt came in, shouted Allahu Akbar and uh the people sitting in there said “bless you”. And so that was the joke.”* (A3,38)

Only after a Muslim student drew attention to the problematic content, other students evaluated the comic as *“wide off the mark”* and to *“serve racist stereotypes”* (A3,38).

Also, the teaching material is subject to criticism. One student remembers that *“these teaching materials are very problematic and discriminating […]. Always for the disgusting diseases it’s racialized people”* (A1,40). Moreover, students criticize the use of racial categorizations in books, lectures, and medical guidelines for falsely implying biological differences between races. One participant claims that when these categorizations come up in lectures for example, in health data from the US-context, they are often not sufficiently contextualized (B5,124). At the same time, students identify institutional racism in what is not taught, for example in the context of teaching common diseases on darker skin in dermatology: *“classic children's diseases […] mumps, measles, rubella, cyanosis are not shown in the textbooks with black people”* (F3,71). Students find this knowledge gap problematic as it may impede the quality of healthcare for patients with darker skin (D1,15). 

#### 3.2. Medical students have difficulties understanding levels of racism

Although the participants report many pertinent experiences, the definite assessment of the range of racism in medicine and healthcare poses a challenge. The medical students lack the theoretical knowledge to define racism since* “everyone defines racism differently or sees the boundaries differently”* (D3,41). Furthermore, some participants have difficulties to differentiate between racism and professional medical reasoning and behaviour. For example, there is intensive discussion about the legitimacy of the term *“Morbus Mediterraneus”* (e.g., A2,24). While some find its use *“super racist”* (A1,25), others understand it as *“very practical”* (A3,33) because *“ultimately this is also very important for us in medicine, of course within a certain limit, […] to classify people and to think in rough pigeonholes. In the end, there's no other way to do it”* (F4,36). Racial categorizations are therefore not understood as* “per se”* racist by some students as it is *“about medical conditions, somehow to represent and to be able to measure certain things”* (F2,38).

Medical students find it difficult to identify structural racism. While it is easier for them to identify racism on the individual level, the understanding of structural racism in the German context poses a significant challenge. Confronted with examples of structural racism in the US-context during the focus groups, students can identify structural disparities but repeatedly speculate to what extent the true reason for health disparities is a matter of lifestyle (C2,64). When discussing similar phenomena in the German context, they tend to play down the effect of structural racism on health compared to other factors, such as socioeconomic status or educational background. One student with no personal experience of racist discrimination reasons: *“I hope that they are not treated badly due to their skin colour. […] but that it [the unequal treatment] is based on the socio-economic status”* (A3,75). While healthcare disparities between different patient groups are acknowledged, racism is not seen as the root cause, but rather substituted for other categories like class.

#### 3.3. Medical students are uncertain about how to deal with experienced racism

When discussing racism in medicine, students with and without own experiences with racism often verbalize insecurities about how to react in any given situation or handle racism in general. Situational and institutional factors are named as possible causes. A first-year student experienced an anti-Asian remark from a general practitioner in an internship and reflects on her lack of reaction: *“In that situation I actually didn't really know how to react there because the atmosphere is so a bit loose and yeah, somehow was a bit weird”* (B6,53). Despite evaluating the remark as racist, the perceived *“loose”* atmosphere made her question her own experience and finally prevented her speaking up. Humour is named as a prominent disguise of racist situations and causes insecurities about how to respond (D5,37). Additionally, the pronounced hierarchical power structures in the medical context are addressed as a factor why medical students do not speak up when faced with racist discrimination: 

*“because you are at the bottom of the food chain anyway […]. And then you usually prefer to think 'I'll accept it now' or 'I'll just look now, I'll look past it', instead of thinking that it really is a racist attack.”* (D3,41)

In this context, sharing experiences with racism might be associated with feelings of vulnerability and insecurity and/or are met with disbelief:* “If you then go to colleagues and they say, 'oh come on, it's not meant like that'. Then you feel somehow totally invalid”*. (D3,41) Students describe it as a *“taboo”* (F3,86) to name racism which results in a* “culture of silence”* (A1,136). In the context of academic teaching, they fear consequences when criticizing lecturers. One student describes the intimidating response of a physiology lecturer after calling out racist content in a lecture as *“a very, very nasty e-mail”* (E1,106). As a result, the student preferred not to continue her studies in the same semester as she did not see any other way to avoid being examined by that lecturer (E1,106). Against the backdrop of such experiences, students call for public institutional contact and counselling points for those who experience racism in medical education (B2,167).

#### 3.4. Medical students call for antiracism in medical education and beyond

The participants unanimously agree that racism in medicine should be made an explicit topic of medical education as it is currently mostly neglected: *“but we didn't talk about racism at all […] in the official curriculum”* (C1,107). Yet, the modalities and contents of antiracism in medical education are discussed controversially. Regarding the modalities, medical students discuss whether classes on racism in medicine should be mandatory or optional. While some see racism as a general societal problem that does not have to be covered in the medical curriculum (F2,92), others recommend mandatory classes: *“design the courses in such a way that you are forced to deal with it […] just to create a basic awareness”* (D4,104). While certain subject areas such as *“medical sociology and psychology”* (B7,162), *“medical ethics”* (C1,107), or *“communication training”* (A3,130) are seen as being particularly responsible to address issues of racism, medical students emphasize that critical approaches to racism should not only form a part of academic teaching. The effectiveness of university medical education is called into question given the power of the *“hidden curriculum”* (A1,80) and *“that these categories and prejudices […] are passed on from generation to generation”* (F2,51). Therefore, medical students stress the need to include critical education on racism in clinical practice and highlight the importance of role models (A1,136). However, they criticize a general lack of expertise on racism in medicine and healthcare in the faculty staff at medical schools in Germany (E5,100).

Regarding the content of antiracist medical education on the one hand, students ask for teaching of specific knowledge, e.g., about the effects of racism on health (F1,93), dermatology on darker skin (E4,91) or the role of academic medicine during racist crimes in the context of German colonialism (C4,54; D3,100). On the other hand, they demand possibilities for self-reflection to develop a critical professional attitude towards racism. It was suggested that this latter point be *“integrated into medical studies, that one talks about how one deals with one’s own racism, how one recognizes it, how one averts it”* (B4,154). In addition, they suggest training that equips them with the necessary skills to react and deal with racist situations in clinical practice: *“how one also reacts to racist situations, with the staff, but also with patients”* (B4,154). Finally, students call upon universities to promote a more diverse student and staff body, because *“representation matters”* (C4,117) and medical students and physicians in Germany are perceived as *“very white, very privileged”* (B4,154).

## 4. Discussion and conclusion

Our results show that medical students discuss racism as a ubiquitous phenomenon in medicine and healthcare. Racism can appear in all medical specialties and interpersonal interactions. They also highlight the difficulties of medical students to identify and evaluate racism, especially at a structural level. Furthermore, students are uncertain how to deal with racism, either when it is experienced or witnessed. They also complain about a lack of anti-discriminatory structures in medical institutions. Finally, our participants see it as the responsibility of medical education to tackle racism in medicine and healthcare.

Our findings show similarities with international studies, e.g., regarding the ubiquity of racism in medicine [17] or the specific challenges to address racism in the medical context [18], such as a widespread naturalistic understanding of races [19], [20], [21]. A qualitative study with medical students on racism in medicine in the US [18] arrived at similar hypotheses on the ubiquity of racism as well as the challenges of addressing racism in medicine, due to the hierarchical structures and power dynamics that are prominent in this field. Therefore, these results from international research may prove useful for the development of German medical education. 

In fact, a multitude of recommendations and evaluations of antiracist curricular interventions have been published in the Anglo-American literature [22], [23], [24], [25], [26], [27], [28], [29], [30]. However, their direct application to German medical education is limited due to various historical, social, and cultural specificities. In the German context, the insufficient theoretical conceptualization of the category “race” and the lack of systematic research on the effects of structural racism on health pose a specific challenge. This is connected to the widespread use of the imprecise category “migration background” in German research, which can also be seen in our results [31]. In Germany, the social sciences and humanities have more developed discourses on racism than medicine. In educational science for example, constructivist conceptions of race as the result of racism [32], [33] are used as the basis to take up didactical approaches for developing antiracism as a professional attitude [34]. Further, approaches from sociology or history, such as understanding the origins of racism in colonialism and racial anthropology, could help to address racism in medicine and healthcare [14], [30], [33], [35], [36]. 

Our study is not exhaustive. Although we included students from all phases of medical studies and a great variety of different medical faculties in Germany, our overall sample is comparatively small. Furthermore, around one third of the participants had been interested in racism in medicine beforehand or were engaged in political activism or had prior knowledge. Further systematic quantitative research is necessary to develop a more nuanced and representative picture. Due to the multiprofessional nature of the healthcare system, the role of racism needs to be studied from the perspectives of other healthcare professions as well. The focus group method might also have influenced the way racism was addressed e.g., by social desirability effects. Finally, it must be stated that our social position as medical ethicists with no personal experience of racism may have also influenced our research perspective [37], [38].

Nevertheless, our findings allow us to draw several conclusions concerning antiracist medical education. The perceived ubiquity of racism in medicine and healthcare implies the necessity to foster antiracist action in a comprehensive way, including changes in both academic and clinical settings on individual and institutional levels. As medical students present difficulties in identifying and discussing racism, there is a need for antiracist education in the academic and clinical settings as well as in their own professional development. This is especially noted with their perceived difficulty to identify structural racism, which suggests the need for teaching on the social determinants of health and the role of structural racist discrimination. The appropriate integration of such aspects into the curricula should also be reflected at the level of quality assurance in medicals education, but also in other health professions (for similar approaches, cf. [39], [40]). Furthermore, institutional support must be systematized to deal with racism both in medical education and in practice. This includes accessible persons of trust but also more substantial changes regarding the perpetuation of racism in the hidden curriculum through hierarchical power structures, institutional taboos, and a weak feedback culture specific to the field of medicine. The diverse range of ideas and suggestions from medical students may prove useful for participative approaches of innovative curriculum development. Overall, our findings stress the importance of antiracism in the mandatory medical curriculum as intended by NKLM 2.0’s learning objective VIII.6-04.4.13 [https://nklm.de/zend/objective/view/id/10001324/essential/yes/lve/211449]. Yet, they also show that didactic approaches are not enough. Ultimately, medical schools also need to address racism comprehensively at an institutional and structural level to foster substantial change. 

## Acknowledgements

We would like to thank Shagana Shanmuganathan and Silke Schicktanz (UMG) for their support in the conception of the study, Houda Hallal (Köln) for her helpful comments on the manuscript. For the exchange on content and methods we thank Tanja Gangarova, Felicia Boma Lazaridou and Hans Vogt (DeZIM). Many thanks to Lucas Rateitschak for his help in the transcription process and to Cai Weaver for his linguistic editing. 

## Competing interests

The authors declare that they have no competing interests. 

## Figures and Tables

**Table 1:**
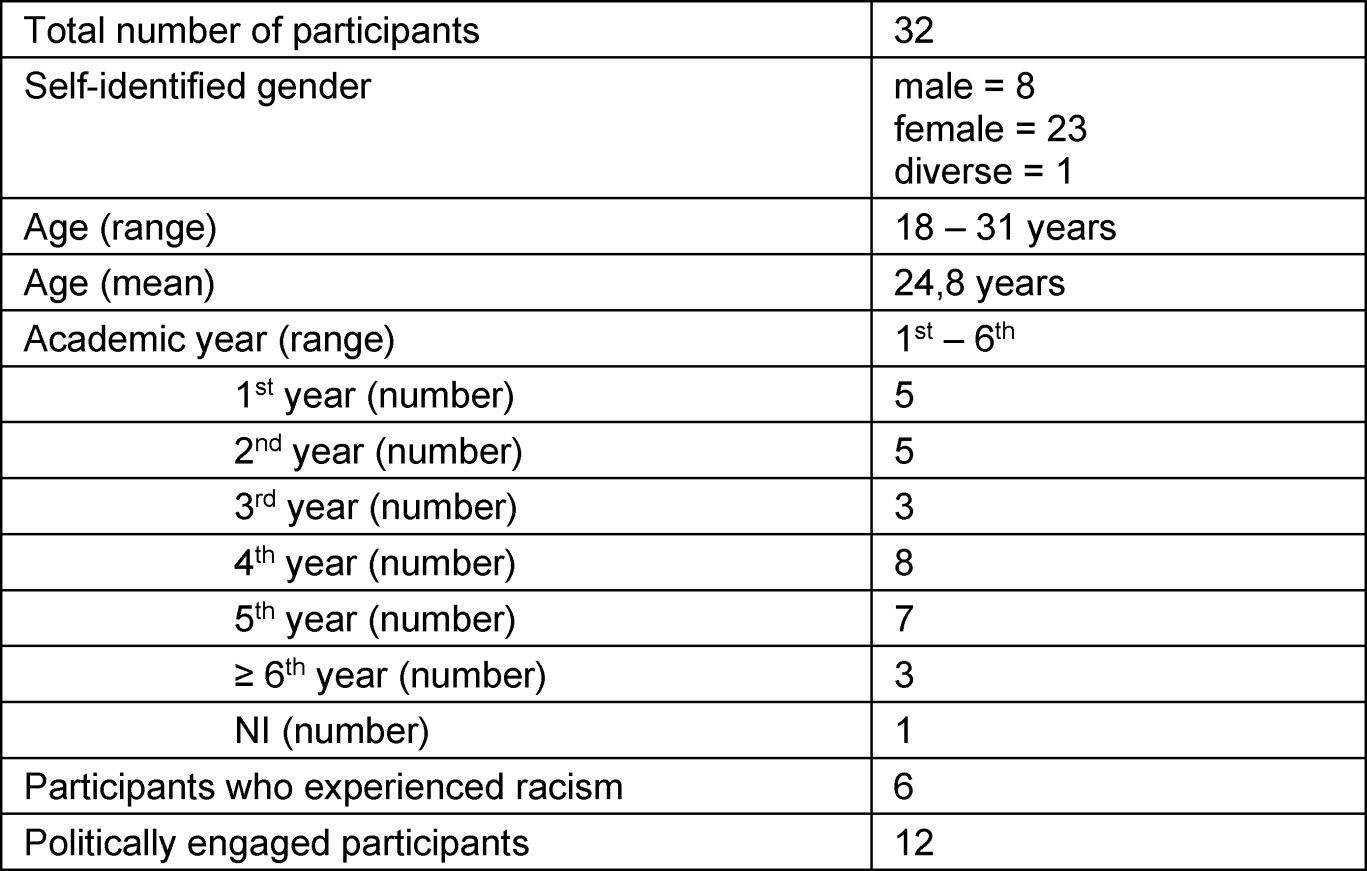
Participants characteristics
